# Acupuncture improves postoperative symptoms of pigmented villonodular synovitis: A protocol for systematic review and meta analysis

**DOI:** 10.1097/MD.0000000000021748

**Published:** 2020-08-14

**Authors:** Hongzhi Tang, Huaying Fan, Fei Luo, Li Huang, Shichuan Liao, Wenjing Yu, Yunbei Chen, Jiao Chen, Xuefei Qin

**Affiliations:** a Outpatient Department of Sichuan Orthopedic Hospital; b The Acupuncture and Tuina School, The 3rd Teaching Hospital, Chengdu University of Traditional Chinese Medicine, Chengdu City, Sichuan Province, China.

**Keywords:** acupuncture, pigmented villonodular synovitis, protocol, systematic review

## Abstract

**Background:**

Pigmented villonodular synovitis (PVNS) is a benign proliferative disease of synovial joint, synovial sac and tendon sheath. PVNS is usually treated by surgery, but postoperative joint dysfunction and pain will be accompanied, which seriously affects the quality of life. The purpose of this review is to evaluate the effectiveness and safety of this intervention in patients with pain and dysfunction caused by postoperative symptoms of PVNS.

**Methods:**

We will search the EMBASE, the Cochrane Library, Ovid MEDLINE, PubMed, Web of Science, Chinese Biomedical Literature Database (CBM), Chinese National Knowledge Infrastructure (CNKI), Wanfang Database, the Chongqing VIP (VIP), the US National Institute of Health, the NIH clinical registry Clinical Trials, the ICTRP, and the Australian New Zealand Clinical Trials Registry and the Chinese clinical registry, from their inception to 1st July 2020. Randomized controlled trials that include patients with postoperative symptoms of pigmented villonodular synovitis receiving acupuncture therapy versus a control group will be included. The selection of studies, data extraction and risk of bias assessment will be conducted by 2 independent researchers. A third review author resolved disagreements. The dichotomous data will be presented as risk ratios with 95% confidence intervals (CIs) and the continuous data will be presented as weighted mean differences or standardized mean differences with 95% CIs. Evidence quality will be evaluated using the GRADE system.

**Results:**

The results will be disseminated through a peer-reviewed journal publication.

**Conclusions:**

This systematic review will provide updated evidence of various types of acupuncture specifically focuses on its effectiveness and safety for patients’ pain and dysfunction caused by post-operation of pigmented villonodular synovitis.

**Ethics and dissemination:**

Ethical approval is not necessary as this review will not require data from individual patients. The results of this will be published through peer-reviewed journal articles or conference presentations.

**Registration:**

Open Science Framework (OSF). 2020, July 7. 10.17605/OSF.IO/CZW9P.

## 1 Introduction

Pigmented villonodular synovitis (PVNS), which is also termed tenosynovial giant cell tumor, is a rare benign proliferative disease of the synovial joint, synovial bursa, and tendon sheath.^[1]^ PVNS is a condition of the synovial membrane that is characterized by the presence of inflammation and hemosiderin deposition in the synovium.^[2]^ At present, there are still controversies about its pathogenic process, inflammation theory and tumor theory are the most common theories.^[3–5]^ PVNS is typically found as a monoarticular process, and can involve any structure lined with synovium. It most often involves the load-bearing joint, especially the knee joint, followed by the hip, ankle, shoulder, and elbow.^[6]^ Histologically, it is characterized by inflammation, hemosiderin deposition, multinucleated giant cells, and apolipoprotein macrophages.^[7]^ The disease most commonly presents in young adults between the ages of 30 and 40 years. PVNS has an estimated incidence of 2 to 8 cases per million per year in the general population, and although some authors have reported male predominance, other authors have reported no difference between sexes3–7.^[3,7–10]^ PVNS can be divided into 2 forms: localized and diffused. Both of these forms are similar histologically, but there are great differences in biological behavior, treatment principles, and prognosis.^[11]^ So far, the key to treatment for localized and diffuse PVNS by complete surgical resection of the diseased synovial membrane, but there is a higher risk of recurrence after the operation, especially in diffuse PVNS. According to reports, the recurrence rate is between 8% and 56%.^[12,13]^ In particular, its long rehabilitation period is related to potential postoperative stiffness or wound complications.^[12]^ All of these will lead to joint dysfunction and pain, seriously affecting the quality of life.

Acupuncture is commonly used for musculoskeletal disorders, and it has been suggested as a meaningful nonsurgical intervention for managing joint pain and dysfunction.^[14]^ The mechanism of acupuncture treatment whas not been fully elucidated. Some studies have pointed out that the mechanism of acupuncture treatment may be related to the local effect, which refers to the release of neuropeptide and other substances by stimulating the sensory nerve endings of skin and muscle tissue. The research shows that acupuncture can promote the release of β-endorphin10^[15]^ and increase the concentration of 5-hydroxytryptamine in brain tissue,^[16]^ so as to reduce the sensitivity of tissue to pain and improve joint activity. Acupuncture can stimulate peripheral mechanoreceptors and inhibit nociceptors to reducing pain, and can enhance exchange between synovial fluid and cartilage matrix to increasing joint mobility.^[17]^

Previous systematic reviews did not evaluate acupuncture for postoperative rehabilitation after PVNS resection. Therefore, we decided to make a systematic review on the basis of the best available evidence and methods to evaluate the effectiveness and safety of acupuncture in the recovery of the joint pain and dysfunction after PVNS resection.

## 2 Methods

### 2.1 Protocol registration

Prospective registration of this study has been approved by the Open Science Framework (OSF) registries (https://osf.io/registries), and the registration number is 10.17605/OSF.IO/CZW9P. The protocol was written following the Preferred Reporting Items for Systematic Reviews and Meta-Analyses Protocols (PRISMA-P) statement guidelines.^[18]^

### 2.2 Criteria for study selection

There will be no restrictions related to setting or location.

### 2.3 Types of studies

We will include randomized controlled trials (RCTs), which improve the joint dysfunction and pain by acupuncture treatment after PVNS resection. We will exclude the quasirandomized, nonrandomized controlled trials, uncontrolled clinical trials (eg, case studies), qualitative studies, and laboratory studies. We will include RCTs, which using single-blind, double-blind or open-label design. For cross-over trials, data will be extracted from the first period only, to avoid potential carryover effects. Study eligibility will not be restricted by language or date of publication.

### 2.4 Types of participants

Our review will include all adult patients (aged ≥18 years) with a definite diagnosis or provisional diagnosis of PVNS: patients with swelling and pain: the first symptoms of all patients were repeated swelling and pain of joints at different levels, after activity aggravate; the basic body temperature of the patient is normal, and the skin temperature of the affected limb is higher than that of the healthy side; reduced range of motion and limited function of affected limb; patients with surgical resection of the diseased synovial membrane, and some pathological tissues were taken for pathological examination: it was confirmed that PVNS.

We will exclude trials that include any patients with a history of rheumatic, tuberculous, infectious and hemophilic knee synovitis, or the patient have other bone and joint diseases or tumor-related diseases, resulting in joint deformity and dysfunction. There will be no restriction on age, sex, and ethnic origin.

### 2.5 Types of interventions

All randomized controlled comparisons of acupuncture will be included. Trials evaluating the primary or add-on effects of acupuncture are eligible.

Acupuncture therapy (acupuncture, auricular acupuncture, electroacupuncture, scalp acupuncture, warm needling, acupoint injection, acupoint catgut embedding, among others) in the treatment group will be considered only involving acupuncture points, pain points, or trigger points, which must be described as acupuncture, whereas the control group will be treated with nonacupuncture therapy (Western medicine, traditional Chinese medicine, conventional treatment, placebo) or sham acupuncture.

We will exclude studies that only compare different forms or methods of acupuncture (eg, transcutaneous electrical nerve stimulation) and compare acupuncture with any complementary and alternative therapies for which the efficacy is not yet established (eg, Chinese herbs).

### 2.6 Types of outcome measures

No studies were excluded on the basis of outcome measure used.

#### 2.6.1 Primary outcomes

1. Pain intensity (measured by validated scales, such as the visual analogue scale or numeric rating scale);
2. Joint function (measured by range of motion).

#### 2.6.2 Secondary outcomes

1. Health-related quality of life measured on validated scales such as Short-Form 36 (SF-36) Health Survey or EuroQoL EQ-5D;
2. Patient global assessment of treatment outcomes, such as the Patient Global Impression of Change (PGIC);
3. Adverse events;
4. Recurrence rate;
5. Recovery time of patients.

### 2.7 Search methods for identification of studies

#### 2.7.1 Electronic searches

From the inception dates to August 1, 2020, the following databases will be searched: EMBASE, the Cochrane Library, Ovid MEDLINE, PubMed, Web of Science, Chinese Biomedical Literature Database (CBM), Chinese National Knowledge Infrastructure (CNKI), Wanfang Database, the Chongqing VIP (VIP). The searching strategy of PubMed is presented in Supplemental Digital Content (Table 1).

**Table 1:**
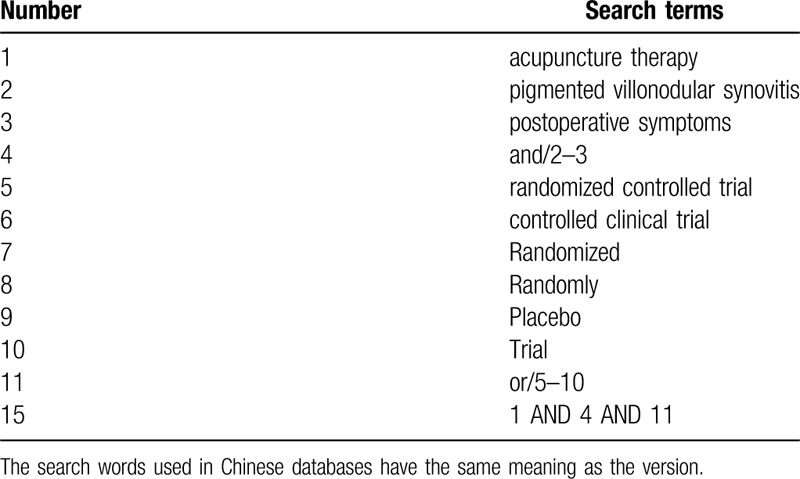
Search strategy used in PUBMED database.

#### 2.7.2 Searching other resources

Ongoing trials with unpublished data will also be retrieved from the following clinical trial registries: the US National Institute of Health, the NIH clinical registry Clinical Trials. The International Clinical Trials Registry Platform (ICTRP), the Australian New Zealand Clinical Trials Registry, and the Chinese clinical registry. Useful but incomplete data will be obtained for data synthesis from the contact trial researcher.

### 2.8 Data collection and analysis

#### 2.8.1 Selection of studies

Two review authors (THZ and FHY) will independently screen all abstracts identified from the literature search, then exclude those that are clearly irrelevant (eg, studies focusing on other conditions, reviews, among others). We will obtain full texts of all remaining references and the same 2 review authors will screen and exclude clearly irrelevant articles according to the selection criteria. We will resolve disagreements by discussion or with a third review author.

#### 2.8.2 Data extraction and management

**Figure 1:**
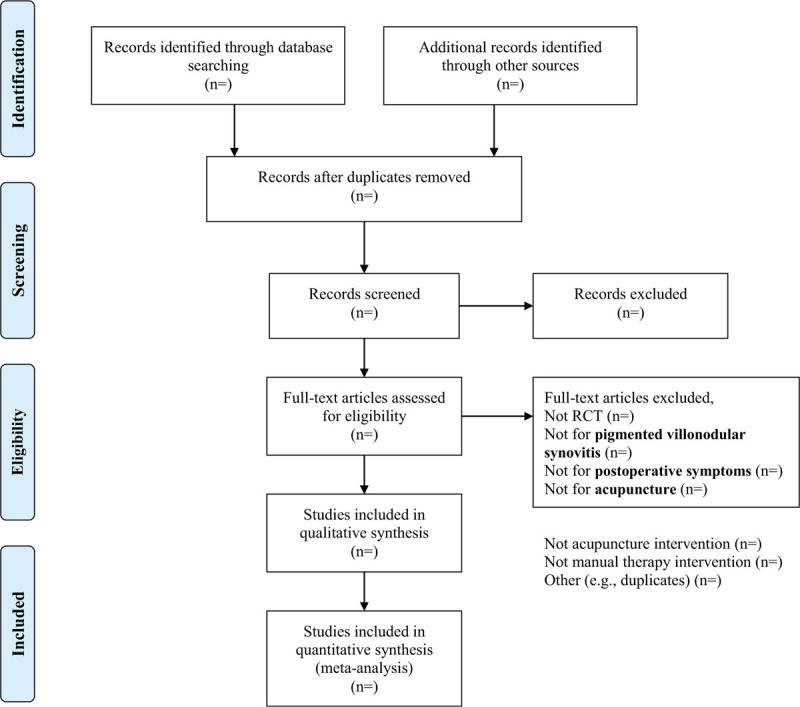
PRISMA flow diagram of study selection.

Two review authors (THZ and FHY) will independently use a specially designed form to extract information on participants, methods, interventions, outcomes, and results. In particular, we will extract first author's name, year of publication, age, sex, duration of disease, sample size, number and type of centers, treated and analyzed, number of reasons for dropouts, duration of baseline, treatment, and follow-up, details of acupuncture treatments (such as selection of points, number, frequency, and duration of sessions), achievement of De-Qi (an irradiating feeling considered to indicate effective needling), number, training, and experience of acupuncturists, details of manual therapy, and the control interventions (sham technique, type, and dosage of drugs). We will contact the first or corresponding authors via email and ask them to provide additional information if necessary. The flow chart based on PRISMA is displayed in Figure 1.

#### 2.8.3 Assessment of risk of bias in included studies

The quality of the included trials will be evaluated by 2 reviewers using the Cochrane Collaboration's tool.^[19]^ Six aspects (randomly generated sequence number, allocation concealment, blinding of participants and personnel, blinding of outcome assessment, incomplete outcome data, selective reporting, and other bias when required) will be assessed. For each aspect, the trial will be rated as high, low risk, or unclear of bias. A trial that is rated as high risk of bias in ≥1 aspects will be graded as “high risk," whereas a low risk of bias in all aspects will be graded as “low risk." If there is a low or unclear risk of bias for all main aspects, the trial will be rated as “unclear risk." The contact person or corresponding author will be contacted if basic information is missing for the risk of bias assessment. The rating results will be cross-checked and discrepancies resolved through discussions and the arbitration of a third reviewer.

#### 2.8.4 Measures of treatment effect

Efficacy data will be synthesized and statistically analyzed by using Rev Man software (Review Manager Version 5.3 for Windows, The Nordic Cochrane Center, Copenhagen). Dichotomous data will be investigated by using a risk ratio with 95% confidence intervals (CIs). For continuous outcomes, data will be analyzed by using a standard mean difference (SMD) with 95% CIs or a weighted mean difference (WMD). The WMD will be used for the same scale or the same assessment instrument; SMD will be used for different assessment tools.

#### 2.8.5 Unit of analysis issues

Different units of analysis will be planned to be subjected to a sensitivity analysis.

#### 2.8.6 Dealing with missing data

We will contact the authors in China via telephone, and authors from elsewhere via email to obtain missing data, if necessary. For all outcomes, we will carry out analyses, and both per-protocol (PP) analysis and intention-to-treat analysis will be accepted. Attrition rates, for example dropouts, losses to follow up, and withdrawals, will be investigated. If the missing data are not accessible, we will exclude these articles and synthesis the rest of the included studies.

#### 2.8.7 Assessment of heterogeneity

According to the Cochrane Handbook for Systematic Reviews of Interventions, assessment of between-trial heterogeneity will be based on visual inspection of the forest plot, and more formally on the *I*^2^ statistic. We define that an *I*^2^ of <40% is low, 30% to 60% is moderate, 50% to 90% is substantial, and 75% to 100% is considerable.^[19]^

#### 2.8.8 Assessment of reporting bias

We will not perform funnel plots to assess the reporting bias due to the lack of sufficient studies (<10).

#### 2.8.9 Data synthesis

We will use Rev Man software (Review Manager Version 5.3 for Windows, The Nordic Cochrane Center, Copenhagen) to carry out statistical analysis. A random-effects model will be used to calculate the pooled effect estimates because substantial clinical heterogeneity is expected among the studies included in this review. If considerable heterogeneity (*I*^*2*^ > 75%) is observed, we will not meta-analyze the trials and will qualitatively synthesize the data.

#### 2.8.10 Subgroup analysis and investigation of heterogeneity

If one of the primary outcome parameters demonstrated statistically significant differences between intervention groups, we will plan to use subgroup analyses. Classifications are as follows:

1. Different joint (ie, hip, elbow, ankle, knee);
2. Different type of acupuncture stimulation (ie, manual, electrical, or other stimulation techniques, such as pharmaco-acupuncture, acupotomy, or thread-embedding therapy).

#### 2.8.11 Sensitivity analysis

We will perform sensitivity analyses to determine whether the results have been influenced by trials where radiologic diagnosis of Rotator cuff tears is not mentioned in the participant eligibility criteria, where measures of variance are missing, and where different methods of analysis are used (random-effects model or fixed-effect model).

### 2.9 Evidence quality evaluation

Two reviewers will use the Grading of Recommendations Assessment, Development and Evaluation (GRADE) system to independently assess the quality of evidence for each outcome. Evidence quality will be rated “high," “moderate," “low," or “very low" according to the GRADE rating standards. The quality of evidence of a specific study will be assessed according to the risk of bias, inconsistency, indirectness, imprecision, publication bias, large effect, dose-response, and all plausible confounding.^[20]^ A summary of findings table will be generated and included in the final report.

### 2.10 Ethics and dissemination

Ethical approval is not necessary as this review will not require data from individual patients. The results of a review provide systematically view and evidence of acupuncture for postoperative symptoms of pigmented villonodular synovitis for clinical practice and further research, and it will be disseminated through peer-reviewed journal articles or conference presentations.

## 3 Discussion

There has not been a systematic study on acupuncture to improve postoperative symptoms of pigmented villonodular synovitis. Acupuncture as a technology is being considered in western medical practice to solve many problems, especially in modern technology or limited or inappropriate effectiveness.^[21]^ In addition, the increasing use of acupuncture around the world has not been mentioned in previous reviews, which means that clinical trials and systematic reviews are necessary to assess the effectiveness and safety of such interventions in patients with pain and dysfunction after postoperation of pigmented villonodular synovitis. This systematic review will provide the latest evidence of various acupuncture treatments, with special attention to their effectiveness and safety in improving postoperative patients with diffuse pigmented villonodular synovitis

## Author contributions

THZ and FHY contributed to the conception and design of the study protocol. The search strategy was developed and run by THZ and LF, who will also screen the title and abstract of the studies after running the search strategy. HL and YWJ will screen full copies of the remaining studies after the title and abstract selection. LF and LSC will extract information from the included studies and enter into the electronic database. QXF will check the accuracy and completeness of the data entry. FHY and CJ will give analysis suggestions during data synthesis. All the authors drafted and revised this study protocol and approved it for publication.

## References

[1] Stephan SR Shallop B Lackman R Pigmented villonodular synovitis: a comprehensive review and proposed treatment algorithm. JBJS Rev 2016;4:10.2106/JBJS.RVW.15.00086 27509331

[2] Tyler WK Vidal AF Williams RJ Pigmented villonodular synovitis. J Am Acad Orthop Surg 2006;14:376–85. 16757677 10.5435/00124635-200606000-00007

[3] Abdul-Karim FW el-Naggar AK Joyce MJ Diffuse and localized tenosynovial giant cell tumor and pigmented villonodular synovitis: a clinicopathologic and flow cytometric DNA analysis. Hum Pathol 1992;23:729–35. 1319390 10.1016/0046-8177(92)90340-9

[4] Ray RA Morton CC Lipinski KK Cytogenetic evidence of clonality in a case of pigmented villonodular synovitis. Cancer 1991;67:121–5. 1985707 10.1002/1097-0142(19910101)67:1<121::aid-cncr2820670122>3.0.co;2-p

[5] Oehler S Fassbender HG Neureiter D Cell populations involved in pigmented villonodular synovitis of the knee. J Rheumatol 2000;27:463–70. 10685815

[6] Myers BW Masi AT Pigmented villonodular synovitis and tenosynovitis: a clinical epidemiologic study of 166 cases and literature review. Medicine (Baltimore) 1980;59:223–38. 7412554

[7] Flandry F Hughston JC McCann SB Diagnostic features of diffuse pigmented villonodular synovitis of the knee. Clin Orthop Relat Res 1994;212–20. 8118978

[8] Flandry F Hughston JC Pigmented villonodular synovitis. J Bone Joint Surg Am 1987;69:942–9. 3597511

[9] Flandry FC Hughston JC Jacobson KE Surgical treatment of diffuse pigmented villonodular synovitis of the knee. Clin Orthop Relat Res 1994;183–92. 8131333

[10] Gu HF Zhang SJ Zhao C A comparison of open and arthroscopic surgery for treatment of diffuse pigmented villonodular synovitis of the knee. Knee Surg Sports Traumatol Arthrosc 2014;22:2830–6. 24474584 10.1007/s00167-014-2852-5

[11] Granowitz SP D’Antonio J Mankin HL The pathogenesis and long-term end results of pigmented villonodular synovitis. Clin Orthop Relat Res 1976;335–51. 770040

[12] Ogilvie-Harris DJ McLean J Zarnett ME Pigmented villonodular synovitis of the knee. The results of total arthroscopic synovectomy, partial, arthroscopic synovectomy, and arthroscopic local excision. J Bone Joint Surg Am 1992;74:119–23. 1463472

[13] Zvijac JE Lau AC Hechtman KS Arthroscopic treatment of pigmented villonodular synovitis of the knee. Arthroscopy 1999;15:613–7. 10495177 10.1053/ar.1999.v15.015061

[14] Birch S Lee MS Alraek T Overview of treatment guidelines and clinical practical guidelines that recommend the use of acupuncture: a bibliometric analysis. J Altern Complement Med 2018;24:752–69. 29912569 10.1089/acm.2018.0092

[15] Sjolund B Terenius L Eriksson M Increased cerebrospinal fluid levels of endorphins after electro-acupuncture. Acta Physiol Scand 1977;100:382–4. 920207 10.1111/j.1748-1716.1977.tb05964.x

[16] Razavi M Jansen GB Effects of acupuncture and placebo TENS in addition to exercise in treatment of rotator cuff tendinitis. Clin Rehabil 2004;18:872–8. 15609842 10.1191/0269215504cr849oa

[17] Bialosky JE Bishop MD Price DD The mechanisms of manual therapy in the treatment of musculoskeletal pain: a comprehensive model. Man Ther 2009;14:531–8. 19027342 10.1016/j.math.2008.09.001 PMC2775050

[18] Shamseer L Moher D Clarke M Preferred reporting items for systematic review and meta-analysis protocols (PRISMA-P) 2015: elaboration and explanation. BMJ 2015;350:g7647. 25555855 10.1136/bmj.g7647

[19] Cumpston M Li T Page MJ Updated guidance for trusted systematic reviews: a new edition of the Cochrane Handbook for Systematic Reviews of Interventions. Cochrane Database Syst Rev 2019;10:ED000142. 31643080 10.1002/14651858.ED000142 PMC10284251

[20] Guyatt GH Oxman AD Schunemann HJ GRADE guidelines: a new series of articles in the Journal of Clinical Epidemiology. J Clin Epidemiol 2011;64:380–2. 21185693 10.1016/j.jclinepi.2010.09.011

[21] Vas J Perea-Milla E Mendez C Acupuncture and rehabilitation of the painful shoulder: study protocol of an ongoing multicentre randomised controlled clinical trial [ISRCTN28687220]. BMC Complement Altern Med 2005;5:19. 16225693 10.1186/1472-6882-5-19 PMC1277817

